# Characterization of stromal vascular fraction and adipose stem cells from subcutaneous, preperitoneal and visceral morbidly obese human adipose tissue depots

**DOI:** 10.1371/journal.pone.0174115

**Published:** 2017-03-21

**Authors:** Karina Ribeiro Silva, Isis Côrtes, Sally Liechocki, João Regis Ivar Carneiro, Antônio Augusto Peixoto Souza, Radovan Borojevic, Clarissa Menezes Maya-Monteiro, Leandra Santos Baptista

**Affiliations:** 1 Post-graduation Program of Medical Clinics, Federal University of Rio de Janeiro – UFRJ, Rio de Janeiro, Brazil; 2 Laboratory of Tissue Bioengineering, National Institute of Metrology, Quality and Technology – INMETRO, Duque de Caxias, Rio de Janeiro, Brazil; 3 Laboratory of Immunopharmacology, Oswaldo Cruz Institute, Oswaldo Cruz Foundation - FIOCRUZ, Rio de Janeiro, Brazil; 4 Nutrology Department, Clementino Fraga Filho University Hospital, Federal University of Rio de Janeiro - UFRJ, Rio de Janeiro, Brazil; 5 Surgery Department, Clementino Fraga Filho University Hospital, Federal University of Rio de Janeiro, Rio de Janeiro, Brazil; 6 Center of Regenerative Medicine, Petrópolis Faculty of Medicine – FASE, Petrópolis, Rio de Janeiro, Brazil; 7 Nucleus of Multidisciplinary Research in Xerem - Biology (Numpex-Bio), Federal University of Rio de Janeiro - Xerém, Duque de Caxias, Rio de Janeiro, Brazil; University of Oslo, NORWAY

## Abstract

**Background/Objectives:**

The pathological condition of obesity is accompanied by a dysfunctional adipose tissue. We postulate that subcutaneous, preperitoneal and visceral obese abdominal white adipose tissue depots could have stromal vascular fractions (SVF) with distinct composition and adipose stem cells (ASC) that would differentially account for the pathogenesis of obesity.

**Methods:**

In order to evaluate the distribution of SVF subpopulations, samples of subcutaneous, preperitoneal and visceral adipose tissues from morbidly obese women (n = 12, BMI: 46.2±5.1 kg/m^2^) were collected during bariatric surgery, enzymatically digested and analyzed by flow cytometry (n = 12). ASC from all depots were evaluated for morphology, surface expression, ability to accumulate lipid after induction and cytokine secretion (n = 3).

**Results:**

A high content of preadipocytes was found in the SVF of subcutaneous depot (*p* = 0.0178). ASC from the three depots had similar fibroblastoid morphology with a homogeneous expression of CD34, CD146, CD105, CD73 and CD90. ASC from the visceral depot secreted the highest levels of IL-6, MCP-1 and G-CSF (*p* = 0.0278). Interestingly, preperitoneal ASC under lipid accumulation stimulus showed the lowest levels of all the secreted cytokines, except for adiponectin that was enhanced (*p* = 0.0278).

**Conclusions:**

ASC from preperitoneal adipose tissue revealed the less pro-inflammatory properties, although it is an internal adipose depot. Conversely, ASC from visceral adipose tissue are the most pro-inflammatory. Therefore, ASC from subcutaneous, visceral and preperitoneal adipose depots could differentially contribute to the chronic inflammatory scenario of obesity.

## Introduction

White adipose tissue has a central role in lipid and glucose metabolism, through production of a large number of hormones and cytokines that modulate of the systemic metabolism [1]. However, the pathological condition of obesity is accompanied by a dysfunctional adipose tissue, with tissue homeostasis disruption due to adipocyte hypertrophy, decreased adipogenesis and angiogenesis [2]. The enhanced abdominal white adipose tissue, rather than the total body adipose tissue, is considered the major predictive feature for the development of a set of metabolic abnormalities known as the metabolic syndrome. The metabolic syndrome increases the risk of type 2 Diabetes and the development of cardiovascular disease [3].

The most commonly defined and studied abdominal white adipose tissues are the subcutaneous and visceral depots, composing the hypodermis and surrounding digestive organs, respectively. Two subdepots can be distinguished in the abdominal subcutaneous depot, the superficial and deep subcutaneous adipose tissue, anatomically separated by the subcutaneous fascial plane [4]. Different visceral abdominal depots can be distinguished in humans: omental adipose tissue, which lines the surface of transverse colon and stomach; mesenteric adipose tissue, located deeper around intestines and retroperitoneal adipose tissue, associated to kidneys in the retroperitoneal compartment [5]. Besides the subcutaneous and visceral tissues, there is the preperitoneal adipose tissue depot [6], a less explored abdominal depot, located between the parietal peritoneum and the transversal fascia macroscopically distinct from the other adipose tissues, including from the deep subcutaneous adipose tissue [7].

Epidemiological data and studies using ultrasonography, magnetic resonance or computed tomography for size estimation of adipose tissue depots, support the idea that an increment in visceral adipose tissue depot (central obesity) represents an increased risk for metabolic disease. On the other hand, obesity characterized by subcutaneous adipose tissue accumulation in gluteo-femoral region and legs (peripheral obesity) is associated with a lower risk [8,9]. Intrinsic biological differences among distinct adipose tissue depots, notably related to their inflammatory profiles, could account for depot-specific contribution to systemic metabolic derangements [10,11]. For example, the obesity-induced macrophage infiltration and accumulation is greater in the visceral adipose tissue than in the subcutaneous one [12] and positively correlates with metabolic syndrome parameters [13]. However macrophage abundance in the two compartments of subcutaneous adipose tissue is distinct, with deep subcutaneous more closely related to the visceral adipose tissue than superficial subcutaneous adipose tissue [14]. Besides, higher distribution of adipose tissue in the superficial compartment seems to have beneficial cardiometabolic effects in patients with type 2 diabetes [4].

Macrophages belong to the adipose stromal-vascular fraction (SVF), together with fibroblasts, endothelial cells, preadipocytes and a population of adult stem cells. In adult organisms, stem and progenitor cells are fundamental for tissue regeneration and homeostasis. They can modulate tissue microenvironment by secreting molecules that exert paracrine effects and by generating new specialized cells [15]. Stem cells are a new paradigm to understand obesity [16] and we have recently shown that the *in vitro* adherent cells from subcutaneous adipose tissue SVF, named adipose-derived stem cells (ASC), are induced into a pro-inflammatory state in morbidly obese subjects. Their ASC have also an impaired lipid accumulation potential, when compared to subcutaneous ASC derived from lean subjects [17].

Many metric, genetic and metabolic studies compared different abdominal white adipose tissue depots, but only a few of them have compared cell composition in human subjects or even the behavior of ASC in obese abdominal adipose tissue depots. Hence, we postulate that subcutaneous, visceral and preperitoneal obese abdominal white adipose tissue depots could have a SVF with distinct composition and ASC with unique properties that could account for different biological properties and contributions to the pathogenesis of obesity. This study will increase our understanding of how adult stem cells from distinct adipose tissue depots participate in the obesity scenario, in order to elucidate the contribution of each depot for the development of obesity.

## Methods

### Patients and tissue harvesting

A small fragment of the subcutaneous, preperitoneal and visceral adipose tissue from the abdominal region was excised from non-diabetic, female, morbidly obese patients (body mass index (BMI) > 40 kg/m^2^) during bariatric surgery (n = 12, age = 37.3 ± 13.4 years, BMI: 46.2 ± 5.1 kg/m^2^). Three of the patients presented systemic arterial hypertension. The Research Ethics Committee of the Clementino Fraga Filho University Hospital, Federal University of Rio de Janeiro, Brazil (Protocol 076/10) approved all the procedures used in this study, and written informed consent was obtained from all the included participants. Data from the subcutaneous adipose tissue have been published in a previous study aiming to compare cells from the same adipose tissue depot (subcutaneous) but from patients with different BMIs [17]. This previous study did not evaluated different adipose tissue depots.

### Isolation of Stromal Vascular Fraction (SVF) and Adipose Stem Cells (ASC) culture

Subcutaneous, preperitoneal and visceral adipose tissue fragments obtained from obese patients were minced and incubated with 1 mg/ml collagenase type II (Sigma Chemical, St. Louis, MO, USA) in a shaking water bath at 37° for 40 minutes. To eliminate mature adipocytes, the obtained suspension was centrifuged (400*g*, room temperature, 15 minutes). The remaining tissue fragments were eliminated through filtration of re-suspended pellets on 100 micrometers strainers. Subsequently, a part of the suspension containing the stromal-vascular cells was evaluated by flow cytometry for SVF subpopulations analysis. Another part of the stromal-vascular cells suspension was plated in tissue culture flasks at a density of 1.2–1.5 x 10^5^ cell/cm^2^ with low-glucose Dulbecco's modified Eagle's Medium (DMEM 1000mg/mL of glucose, LGC, Cotia, Brazil) supplemented with 10% fetal bovine serum (FBS, Cultlab, Campinas, Brazil), 100U/ml penicillin and 100μg/ml streptomycin, for ASC isolation by plastic adherence. Non-adherent cells were washed 24 hours later with phosphate-buffered saline and adherent cells were maintained at 37°C in a humid atmosphere with 5% CO_2_ until the monolayer of cells reached confluence, with medium change every 3–5 days. After confluence, adherent cells were harvested with 0.125% trypsin (Gibco BRL, Rockville, MD, EUA) and 0.78 mM EDTA (Gibco). This adherent cells, named adipose derived stem cells (ASC) were assessed for mycoplasma contamination using a PCR-based assay, performed using the VenorGeM^®^ Mycoplasma Detection Kit (Sigma), according to the manufacturer's instructions. Besides, ASC were analyzed by flow cytometry or maintained in culture for lipid accumulation induction and secretory analyses.

### Flow cytometry assays of SVF cells and ASC

Flow cytometry was performed to analyze the surface marker expression of SVF cells and ASC obtained from the subcutaneous, pre-peritoneal and visceral adipose tissues of the obese patients, as described in detail previously for subcutaneous depots [17]. The flow cytometry analyses were done using FACS Diva 8.0 software, in 100.000 and 20.000 events acquired from SVF and ASC samples, respectively.

According to Zimmerlin and cols [18], we identified pericytes, supra-adventitial and endothelial progenitor cells among non-hematopoietic cells (CD45 negative) in SVF samples. The expression of CD14 and CD206 (mannose receptor) were used to identify resident adipose tissue macrophages among the hematopoietic cells (CD45 positive) [19]. SVF cell quantification was expressed as a percentage of cells obtained among CD45^+^ or CD45^-^ cells. ASC were evaluated according to the expression of CD34, CD146 and the in vitro mesenchymal markers CD105, CD73, CD90.

### Lipid accumulation stimulus

Induction of lipid accumulation and evaluation of the secretion of induced cells were performed as described in detail previously [17]. Briefly, 2×10^4^ ASC were seeded per well of a 48-well plate (in triplicate) and maintained in lipid accumulation inductive media for up to 3 weeks (DMEM with 10% FBS, 10μM insulin, 0.5mM isobutylmethylxanthine, 1μM dexamethasone, and 200μM indomethacin, all from Sigma). Cultures were then maintained in 0.220 ml/well of DMEM with 2% FBS and incubated for 24 hours, after which culture′s supernatant was collected and frozen at -80°C.

Intracellular lipid accumulation was assessed by staining induced cultures with Oil Red O as previously described [17]. Briefly, cultures were fixed in 10% buffered formaldehyde and stained with 5mg/ml of Oil Red O (Sigma) in 70% ethanol for 1 hour, followed by over-staining washing and Oil Red O elution with isopropanol for 10 minutes. The absorbance of the eluted stain was measured in a spectrophotometer at 490nm. The values obtained from the non-induced cultures were subtracted from those obtained in the induced cultures. Three replicates of cultures from each adipose tissue depot (subcutaneous, preperitoneal and visceral) of three patients were analyzed in independent experiments.

### Cytokine secretion by ASC and induced ASC

A multiplex immunoassay was performed to quantify the release of cytokines, chemokines and growth factors in the conditioned medium of cells. Control and induced ASC (after 3 weeks of induction) were incubated with DMEM with 2% FBS for 24 hours. Supernatant of cell cultures was collected from each replicate individually and frozen at −80°C. By the day of analysis, samples were thawed on ice and prepared according to the manufacturer’s recommendations. Bio-PlexPro^™^ human cytokine 17-plex and Human Adipocyte Panel (Millipore) were used to detect IL-1β, IL-2, IL-4, IL-5, IL-6, IL-7, IL-8, IL-10, IL-12(p70), IL-13, IL-17, G-CSF, GM-CSF, IFN-γ, MCP-1, MIP-1β, TNF-α, leptin, adiponectin, resistin, and PAI-1. In three independent experiments, each with one different patient, three replicates of cultures from each adipose tissue depot (subcutaneous, preperitoneal and visceral) were analyzed.

### Statistical analysis

Comparisons among cells from the subcutaneous, preperitoneal and visceral adipose tissue depots were carried out using a paired non-parametric one-way analysis of variance test followed by Dunn’s post-test. Results in graphs were expressed as scatter plots with values from different cell samples harvested from the same patients connected with lines. *p* values less than 0.05 were considered statistically significant. Statistical analyses were performed using the software GraphPad Prism 5.0 (GraphPad Software, La Jolla, EUA).

## Results

### Abdominal white adipose tissue depots from morbidly obese patients exhibited differences in stromal vascular fraction subpopulations

**Fig 1:**
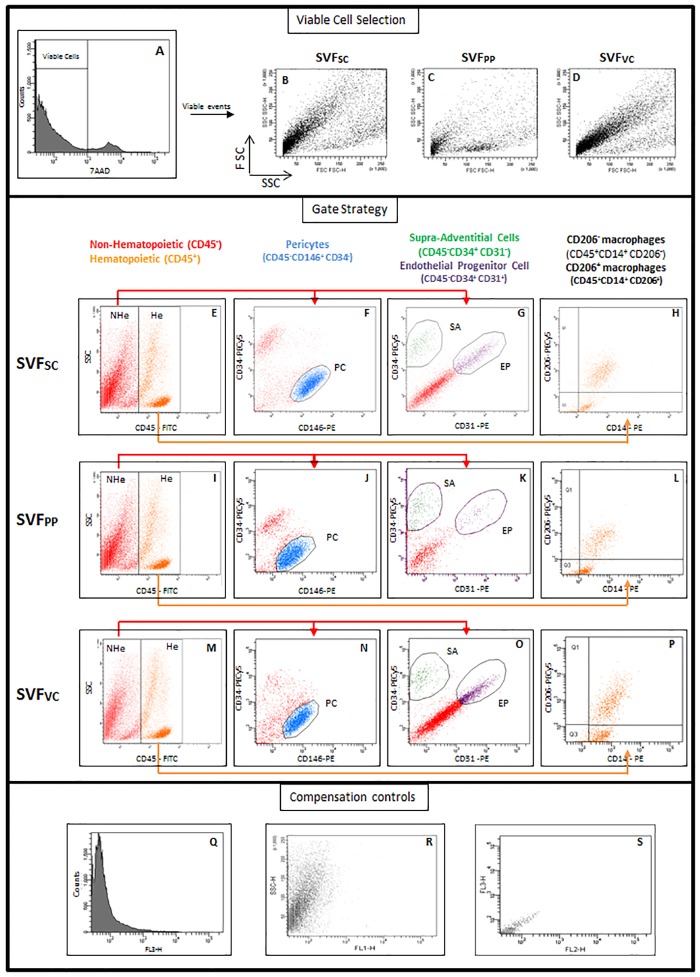
Perivascular and hematopoietic populations classified for analytical flow cytometry. SVF of subcutaneous, preperitoneal and visceral adipose tissues from morbidly obese patients was analyzed by flow cytometry. First, viable cells were identified by 7AAD exclusion (A). Second, viable cells were distributed in a Forward versus Side Scatter plot (B, C, D) and further analyzed according to CD45 expression (E, I, M). The gates were set on non-hematopoietic (CD45neg—NHe) and hematopoietic (CD45pos—He) cells. Third, CD45neg cells (red) were analyzed for CD34 and CD146 expression (F, J, N) or CD34 and CD31 expression (G, K, O). A blue gate was set on the CD146posCD34neg cells to identify the pericytes. A subset of supra-adventitial cells (SA), which were CD34posCD31neg, was identified (green gate) whereas the endothelial progenitor cells (EP) were identified as CD34posCD31pos (purple gate). Among the He cells, two monocyte-macrophage populations were identified (H, L, P): adipose tissue resident macrophages were identified as CD14posCD206pos cells (upper right quadrant) and a population of CD14posCD206neg cells (lower right quadrant). (Q-S) Compensation controls of fluorescence detection. SVF: stromal vascular fraction; SC: Subcutaneous; PP: preperitoneal; VC: visceral; He: hematopoietic; NHe: non-hematopoietic; PC: pericytes; SA: supra-adventitial cells; EP: endothelial progenitor cell.

To evaluate the distribution of SVF subpopulations in different abdominal adipose tissue depots, samples of subcutaneous, preperitoneal and visceral adipose tissues from morbidly obese patients were analyzed by flow cytometry. A representative sample of each depot analyses is shown in Fig 1. 7AAD staining distinguishes non-viable cells present in tissue digests (Fig 1A). Viable cells (negative for 7AAD) of each adipose tissue depot are shown in Fig 1B–1D in a forward versus side scatter plot. Viable cells were then analyzed for the presence of the classifying markers (CD45, CD146, CD34, CD31, CD14, and CD206) previously described [17–19]. The pan-hematopoietic marker CD45 distinguishes hematopoietic (He) and non-hematopoietic (NHe) cells (Fig 1E, 1I and 1M). The NHe cells (CD45neg) contained pericytes (PC; CD146posCD34neg; Fig 1F, 1J and 1N), supra-adventitial cells (SA; CD34posCD31neg; Fig 1G, 1K and 1O), endothelial progenitor cells (EP; CD34posCD31pos; Fig 1G, 1K and 1O) [18].

The monocyte-macrophage CD14pos populations were analyzed among the hematopoietic cells (He), according to the expression of CD206 [19] (Fig 1H, 1L and 1P). Two mono-macrophage subpopulations were identified: CD14posCD206pos cells and CD14posCD206neg cells.

**Fig 2:**
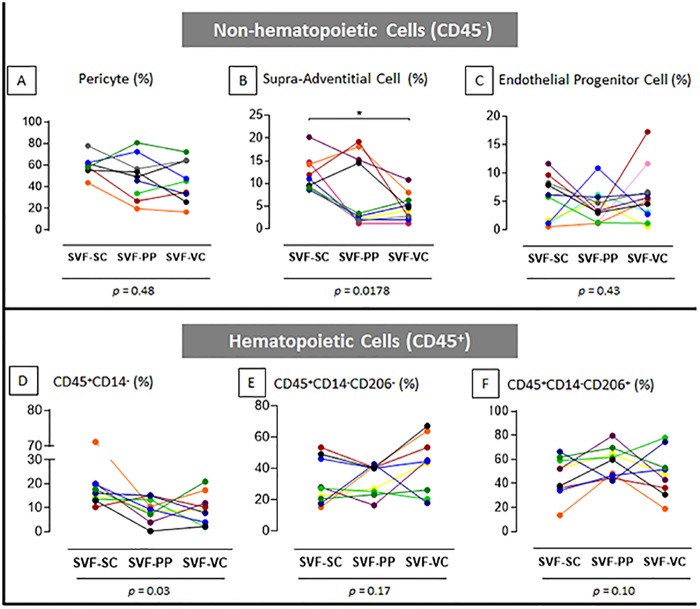
SVF contents of subcutaneous, preperitoneal and visceral adipose tissues from morbidly obese patients. The quantitative data of the flow cytometry analysis shows the distribution of the non-hematopoietic (A, B, C) and hematopoietic (D, E, F) cell subpopulations. The percentage of each subpopulation analyzed from each patient is expressed. Values obtained from different cell samples harvested from the same patient are connected with lines. *p* values under graphs resulted from statistical tests. Asterisk represents the *p* value from the post-test of statistical analyses: (*) p <0.05. n = 9 to 11, according to the population. SVF: stromal vascular fraction; SC: Subcutaneous; PP: preperitoneal; VC: visceral; AT: adipose tissue.

No differences were observed in pericytes (PC; CD146posCD34neg) or endothelial progenitor cells (EP; CD34posCD31neg) content among the studied depots (Fig 2A and 2C). However, the subcutaneous depot was the most enriched with supra-adventitial cells (SA; CD34posCD31neg; Fig 2B, *p* = 0,01). Additionally, the percentage of CD45posCD14neg population differs among depots (Fig 2D, *p* = 0,03), but no significant differences were identified for CD206neg macrophages or CD206pos macrophages (Fig 2E and 2F).

### ASC from distinct abdominal adipose tissue depots are morphologically and phenotypically similar, but exhibit remarkable differences in cytokine secretion

**Fig 3:**
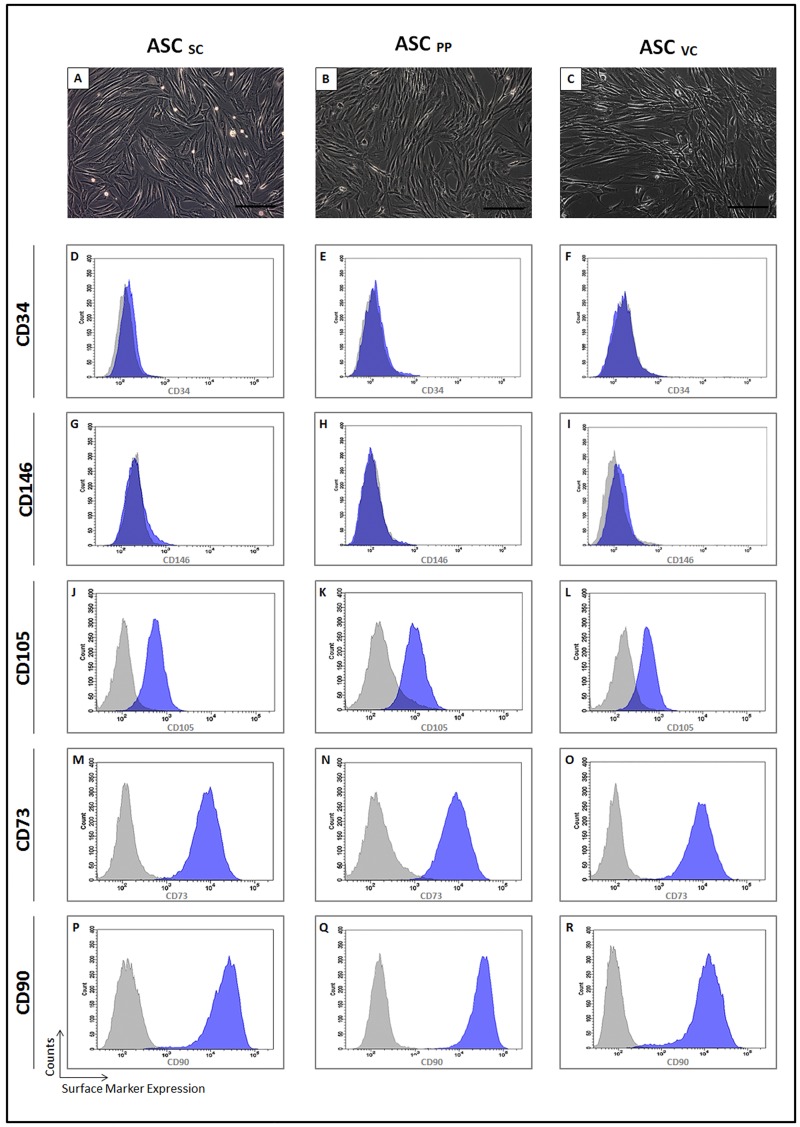
Morpho-phenotypical characterization of ASC from the subcutaneous, preperitoneal and visceral adipose tissues of morbid obese patients. ASC from the subcutaneous (A), preperitoneal (B) and visceral (C) showed fibroblastic cells. (A, B, C) phase contrast. Bar size: 100 micrometers. The histograms show the ASC surface expression of CD34 (D-F), CD146 (G-I); CD105 (J-L); CD73 (M-O) and CD90 (P-R). The histograms of the ASC from the subcutaneous (D, G, J, M, P), preperitoneal (E, H, K, N, Q) and visceral (F, I, L, O, R) depots are representative of independent experiments (n = 3). Gray histogram—negative control; blue histogram—expression of the indicated CD. ASC: adipose stem cell; SC: Subcutaneous; PP: preperitoneal; VC: visceral; CD: cluster of differentiation.

We hypothesized that ASC derived from different anatomical sites in the abdominal region of obese patient could have distinct morphophenotipic characteristics and cytokine secretion profile. After *in vitro* expansion, ASC from the subcutaneous, preperitoneal and visceral adipose tissues showed a typical ASC fibroblastoid morphology (Fig 3A–3C). ASC from the three different depots had a homogeneous expression of CD34 (Fig 3D–3F), CD146 (Fig 3G–3I) and of the *in vitro* mesenchymal markers CD105 (Fig 3J–3L), CD73 (Fig 3M–3O) and CD90 (Fig 3P–3R).

**Fig 4:**
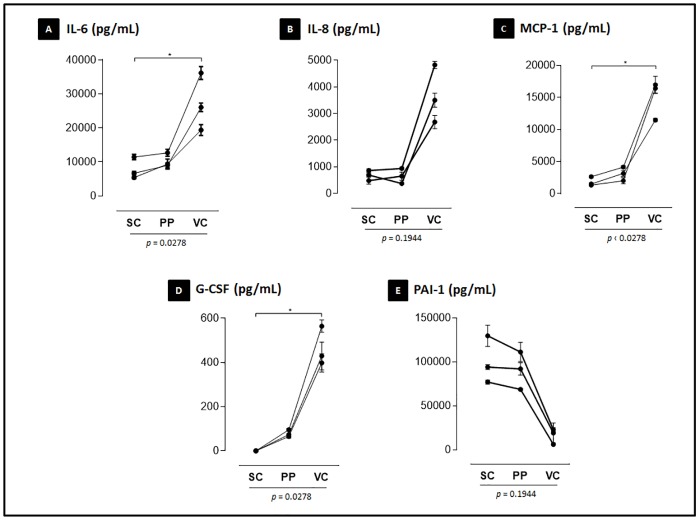
Differential cytokine secretion of ASC derived from distinct abdominal white adipose tissue depots. The concentration of cytokine secreted in the cell culture supernatant quantified using a multiplex assay is expressed as the mean ± SEM (A-E). *p* values under graphs resulted from statistical tests. The asterisks represent the *p* values from the statistical post-tests: (*) p <0.05. Assays were performed in triplicates for each of the 3 patients evaluated. SC: Subcutaneous; PP: preperitoneal; VC: visceral; IL: interleukin; MCP-1: Monocyte Chemoattractant Protein 1; G-CSF: Granulocyte colony-stimulating factor; PAI-1: Plasminogen activator inhibitor-1.

The secretion of ASC was evaluated in the supernatant of highly confluent cultures maintained under standard cell culture conditions. ASC under control conditions from all adipose tissue depots secreted IL-6, IL-8, MCP-1, G-CSF and PAI-1 (Fig 4). Other evaluated molecules were not present at detectable levels and included IL-1β, IL-2, IL-4, IL-5, IL-7, IL-10, IL-12(p70), IL-13, IL-17, GM-CSF, IFN-γ, MIP-1β, TNF-α, leptin, adiponectin and resistin. The ASC from the visceral depot secreted the highest levels of IL-6, MCP-1 and G-CSF (p<0.05). They showed a tendency to secrete the highest levels of IL-8, although there were no statistical differences for this cytokine among depots. Conversely, PAI-1 had a tendency to be more secreted by ASC derived from subcutaneous and preperitoneal depots.

### Preperitoneal adipose tissue contains ASC with the highest adiponectin secretion levels after lipid accumulation stimulus

**Fig 5:**
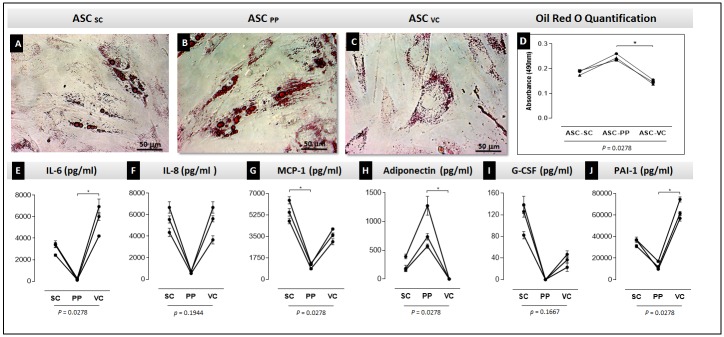
Differential lipid accumulation capacity and cytokine secretion of induced ASC derived from subcutaneous, preperitoneal and visceral depots. The ASC were cultured under lipid accumulation stimulus for up to 3 weeks. Lipid accumulation was analyzed by Oil Red O staining (A-C). The total Oil Red O dye captured was measured in a spectrophotometer. The Graph (D) shows the quantitative results of the Oil Red O staining. ANOVA tests comparing the absorbance were performed. Cytokine secretion in the supernatant of the induced cells is expressed in graphs (E) IL-6, (F) IL-8, (G) MCP-1, (H) adiponectin, (I) G-CSF, (J) PAI-1. *p* values under graphs resulted from statistical tests. The asterisks represent the *p* values from the statistical post-tests: (*) p <0.05. (A-C) Optical microscopy—bar size: 50 micrometers. Assays were performed in triplicates for each of the 3 patients evaluated. ASC: adipose stem cell; SC: subcutaneous; PP: preperitoneal; VC: visceral; IL: interleukin; MCP-1: Monocyte Chemoattractant Protein 1; G-CSF: Granulocyte colony-stimulating factor; PAI-1: Plasminogen activator inhibitor-1.

It was then evaluated the capacity of ASC from the three obese adipose tissue depots to accumulate lipids *in vitro*. ASC from all adipose tissues accumulated few intracytoplasmic lipid droplets after 3 weeks of stimulus and no full differentiation towards adipocytes was observed. The ASC from visceral depot showed the lowest lipid accumulation capacity, while preperitoneal ASC had the highest one (Fig 5A–5D, *p* = 0.0278).

The secretion of ASC was also evaluated after *in vitro* induction of lipid accumulation (Fig 5E–5J). Interestingly, induced preperitoneal ASC showed the lowest secreted levels of IL-6, MCP-1 and PAI-1 (p<0.05), and a tendency to secrete less IL-8 and G-CSF. Conversely, the anti-inflammatory cytokine adiponectin was highly secreted by these cells (p = 0.0278). The adiponectin secretion was not detected in visceral ASC after induction to accumulate lipids (Fig 5H). Other evaluated molecules were not present at detectable levels and included IL-1β, IL-2, IL-4, IL-5, IL-7, IL-10, IL-12(p70), IL-13, IL-17, GM-CSF, IFN-γ, MIP-1β, TNF-α, leptin and resistin.

## Discussion

In the present study, we observed particular properties of ASC from subcutaneous, visceral and preperitoneal adipose tissues that could contribute to the chronic inflammation scenario of obesity. To our knowledge, it is the first time that the SVF composition and cytokine secretion of ASC derived from the preperitoneal adipose tissue are described.

It is hypothesized that functional differences among adipose tissue depots is a consequence of intrinsic characteristics of resident cells of each depot. It was proposed that the adipose tissue SVF contributes to major differences between subcutaneous and visceral adipose depots rather than the adipocyte fraction [20]. We have recently shown that the SVF of the subcutaneous adipose tissue of morbidly obese women is enriched in CD45posCD14posCD206neg population [17]. Accumulated macrophages in obese adipose tissue are associated with inflammation and metabolic complications [13, 21–23]. In the present study, adipose tissue depots from different anatomical sites showed differences in the CD45posCD14pos population (p = 0.03). However, there were no significant differences in the percentage of the CD206pos and CD206neg phenotypes evaluated neither among adipose tissue depots. It has been previously reported no significant differences in CD206pos macrophage counts found in adipose tissue subcutaneous and visceral biopsies in morbid obese women, evaluated by immunohistochemistry analysis [24].

We have found that subcutaneous adipose tissue SVF had the highest content of CD45negCD146negCD34posCD31neg supra-adventitial cells. These cells dwell in the external layer of blood vessels wall as functional preadipocytes [18]. From our results, no remarkable frequency in SVF subpopulations was found in preperitoneal adipose tissue.

We have recently published that the ASC from morbidly obese subcutaneous adipose tissue are in a pro-inflammatory state and have an impaired lipid accumulation potential, when compared to subcutaneous ASC derived from lean subjects [17]. In this study we postulated that inherent properties of ASC may account for the biological specificity among subcutaneous, visceral and preperitoneal adipose tissues and can pave the way for the elucidation of innate pathophysiological roles of each adipose tissue depot during obesity development. In the present study, ASC from the three depots revealed similar fibroblastic morphology. No changes related to surface markers of mesenchymal lineage were found, in accordance with previous studies [25,26]. Considering this homogeneity, multiplex functional assays with ASC were performed with three patients.

Some studies have compared cytokines and chemokines release from whole subcutaneous and visceral adipose tissues, indicating a pro-inflammatory profile of the visceral depot, from both lean and obese subjects [27–29]. A few studies have described cytokines and chemokines synthesized by subcutaneous and visceral ASC [26,30]. Considering mRNA production, the visceral adipose tissue ASC showed the highest levels of inflammatory cytokines as compared to the subcutaneous one [25]. In the present study, we found that ASC cytokine secretion occurred as an adipose tissue depot dependent manner. The visceral adipose tissue ASC was the most pro-inflammatory, secreting the highest levels of the pro-inflammatory cytokines like IL-6 and a tendency to have the highest capacity to secrete IL-8, as expected. It was described that the whole adipose tissue visceral depot secretion of IL-6 is three times higher than subcutaneous depot [27]. We also observed that IL-6 secretion of visceral ASC was almost three times higher than secretion of subcutaneous and preperitoneal ASC. Thus, visceral ASC *in vivo* counterparts could represent a predominant source of these cytokines, contributing to the depot inflammatory characteristic.

MCP-1 and G-CSF were also highly secreted by visceral ASC. Zhu and co-workers^30^ have recently described similar results using a label-free quantitative proteomics approach. We found that ASC from preperitoneal adipose tissue secrete higher levels of MCP-1 as compared to the subcutaneous depot. MCP-1 production is higher in SVF as compared to adipocytes and, among adipose tissue depots, the visceral one shows the highest production [31]. It recruits monocytes, leukocytes, and other inflammatory cells in response to an inflammatory challenge [32]. Elevated levels of MCP-1 may also recruit adipogenic progenitors from the circulation [33] contributing for tissue expansion during obesity. G-CSF is another classical chemoattractant bone marrow hematopoietic cells, mobilizing them to the systemic circulation [34]. G-CSF released by preperitoneal ASC was detected, while no secretion by subcutaneous ASC was observed. Thus, preperitoneal ASC in vivo counterparts could recruit more adipose progenitors and hematopoietic cells from the bone marrow than subcutaneous ASC, while visceral ASC could have the highest recruitment ability.

ASC from visceral adipose tissue revealed the lowest level of PAI-1 release, while subcutaneous and preperitoneal ASC presented a three to four times higher level. High levels of circulating PAI-1 are associated with visceral adipose tissue accumulation, insulin resistance and ischemic heart disease [35]. However, PAI-1 is also associated to cellular migration, angiogenesis [36] and fibrinolysis regulation, contributing for tissue remodeling. This may suggest a higher remodeling capacity, in terms of cellular and extracellular matrix components, of subcutaneous and preperitoneal ASC, as compared to the visceral ones.

There is no consensus about subcutaneous and visceral ASC lipid accumulation potential. While some studies showed similar potential between ASC from these two depots [37,38], others revealed the subcutaneous ASC had a higher potential [39,40]. Differences in induction protocols and methods of analysis may account for this discrepancy. Only one study evaluated preperitoneal ASC lipid accumulation potential, which differentiated earlier than subcutaneous ASC, with no comparison with visceral ASC [41]. In the present study, ASC from the three adipose tissue depots accumulated few intracytoplasmic lipid droplets *in vitro*. ASC from the preperitoneal revealed the highest lipid accumulation potential while those from the visceral revealed the lowest one. Differences regarding the lipid accumulation potential of ASC derived from different adipose tissue depots will be better evaluated with molecular characterization of stimulated cells after long term induction for a full adipogenic differentiation.

Adiponectin secretion was detected only after cytoplasmic lipid accumulation, but not in non-induced ASC, reinforcing the idea that only cells induced to accumulate lipids could be able to secrete adiponectin. Adiponectin was described as being almost exclusively synthesized and secreted by adipocytes and its mRNA is induced over 100-fold during adipogenesis in 3T3-L1 cells [42]. Furthermore, the lowest lipid accumulation capacity of visceral ASC observed in the present study may account for no detectable levels of adiponectin by visceral induced ASC.

Adiponectin is recognized by its anti-inflammatory properties [43,44] and can reverse insulin resistance in obese animals [45,46]. In addition, high levels of adiponectin secretion are associated with low risk of diabetes mellitus II and cardiovascular disease development [47,48]. While preperitoneal ASC induced to accumulate lipids revealed the highest level of adiponectin secretion and the lowest levels of the pro-inflammatory cytokine IL-6, those from visceral depot showed no detectable levels of adiponectin release and the highest level of the pro-inflammatory cytokines.

The cohort of patients included in this study may not necessarily represent the general population in terms of adiposity and socio-economic characteristics, which may limit the application of our study to the whole population. Furthermore, although SVF analyses were performed with the total cohort of the study, ASC functional assays were done with 3 of the patients included in the initial cohort and could represent a limitation. Transcriptomes and epigenetic assays are necessary in order to deepen our comprehension about molecular mechanisms responsible for subcutaneous, visceral and preperitoneal ASC particular properties. Such information will be useful in deciphering the contribution of each adipose tissue depot for chronic inflammation scenario established during obesity development.

Taken together, our results show that ASC from subcutaneous, visceral and preperitoneal adipose depots could differentially contribute to the chronic inflammation scenario of obesity. Since ASC are the main source of cells responsible for adipose tissue homeostasis, it is reasonable to assume not only a pleiotropic role for ASC in the vicious cycle of chronic inflammation [49], but also distinct ASC contributions, in an adipose-depot dependent manner. Differences in ASC behavior can be found not only in different pathophysiological conditions and BMI [17], but also in different abdominal adipose tissue depots in the same obese patient. Preperitoneal adipose tissue revealed the less pro-inflammatory properties, although it is an internal adipose depot. Conversely, ASC from visceral adipose tissue are the most pro-inflammatory. In this context, our study paves the way for future research regarding distinct adipose tissue depots for elucidating cellular mechanisms in obesity.
